# Identifying hepatocellular carcinoma patients with survival benefits from surgery combined with chemotherapy: based on machine learning model

**DOI:** 10.1186/s12957-022-02837-2

**Published:** 2022-12-01

**Authors:** Jie Hu, Ni Gong, Dan Li, Youyuan Deng, Jiawei Chen, Dingan Luo, Wei Zhou, Ke Xu

**Affiliations:** 1 Department of Gastrointestinal Surgery, The Third Xiangya Hospital of Central South University, Changsha, Hunan China; 2 Department of Nursing, The Third Xiangya Hospital of Central South University, Changsha, Hunan China; 3 Department of General Surgery, The Central Hospital of Xiangtan City, Xiangtan, Hunan China; 4 Department of Rehabilitation, The Central Hospital of Xiangtan City, Xiangtan, Hunan China; 5 Department of Hepatobiliary and Pancreatic Surgery, Affiliated Hospital of Qingdao University, Qingdao, China; 6 Clinical Medical College, Chengdu Medical College, Chengdu, Sichuan China; 7 Department of Radiology, The First Affiliated Hospital of Chengdu Medical College, Chengdu, Sichuan China; 8 Department of Oncology, The First Affiliated Hospital of Chengdu Medical College, Chengdu, Sichuan China; 9 Key Clinical Specialty of Sichuan Province, Chengdu, Sichuan China

**Keywords:** Hepatocellular carcinoma, Machine learning, Prognosis, SEER, Chemotherapy

## Abstract

**Background:**

Hepatocellular carcinoma (HCC) is still fatal even after surgical resection. The purpose of this study was to analyze the prognostic factors of 5-year survival rate and to establish a model to identify HCC patients with gain of surgery combined with chemotherapy.

**Methods:**

All patients with HCC after surgery from January 2010 to December 2015 were selected from the Surveillance, Epidemiology, and End Results (SEER) database. Univariate and multivariate logistic analysis were used to analyze the prognostic factors of patients, and the risk prediction model of 5-year survival rate of HCC patients was established by classical decision tree method. Propensity score matching was used to eliminate the confounding factors of whether to receive chemotherapy in high-risk group or low-risk group.

**Results:**

One-thousand six-hundred twenty-five eligible HCC patients were included in the study. Marital status, *α*-fetoprotein (AFP), vascular infiltration, tumor size, number of lesions, and grade were independent prognostic factors affecting the 5-year survival rate of HCC patients. The area under the curve of the 5-year survival risk prediction model constructed from the above variables was 0.76, and the classification accuracy, precision, recall, and F1 scores were 0.752, 0.83, 0.842, and 0.836, respectively. High-risk patients classified according to the prediction model had better 5-year survival rate after chemotherapy, while there was no difference in 5-year survival rate between patients receiving chemotherapy and patients not receiving chemotherapy in the low-risk group.

**Conclusions:**

The 5-year survival risk prediction model constructed in this study provides accurate survival prediction information. The high-risk patients determined according to the prediction model may benefit from the 5-year survival rate after combined chemotherapy.

**Supplementary Information:**

The online version contains supplementary material available at 10.1186/s12957-022-02837-2.

## Introduction

Liver cancer ranks the fourth in the mortality of malignancy in the world, accounting for about 782,000 deaths each year, of which 85% are hepatocellular carcinoma (HCC) [1]. At present, surgical treatment is the most important curative treatment for patients with HCC, but the recurrence rate after 5 years is more than 50%, and the overall 5-year survival rate is only 18% [2, 3]. So, how can we reduce postoperative recurrence and improve postoperative survival in HCC patients? Recently, adjuvant therapy has been shown to improve survival in patients after HCC surgery. In a study of 200 patients with postoperative HCC, the researchers found that adjuvant transarterial chemoembolization significantly improved disease-free survival in patients with tumor size > 5 cm [4]. In a systematic review of 277 patients after HCC surgery, adjuvant immunotherapy was found to reduce the recurrence rate of the disease [5]. There were also some trials found that antiviral therapy could improve the prognosis of patients with HBV or HCV after HCC surgery [6, 7]. However, the benefit object of the adjuvant therapy is not clear yet, and the indication of adjuvant therapy is still controversial. It can be recognized that how to accurately predict the prognosis and rationally identify patients for adjuvant therapy are important issues that we need to explore in the next step.

In terms of survival prediction of patients with HCC after surgery, a large number of predictive indicators have been explored. However, serum *α*-fetoprotein (AFP) remains the unique indicator for postoperative prognosis prediction and follow-up in clinical practice, although its predictive efficiency is also limited [8, 9]. The most effective way to improve the accuracy of prediction is to combine multiple indicators and construct prediction model. In this study, in order to establish the classification, we used the decision tree model, which is a prediction tool that uses classification and numerical data to assign samples to specific categories. Unlike models such as artificial neural networks (ANN), threshold and category predictions calculated by decision tree models often have practical explanations that can be used to provide clinicians with intuitive decisions [10]. At the same time, the decision tree model is especially suitable for the small sample of database. Recently, it has been gradually incorporated into tumor staging because it can use selected factors to classify patients into subgroups with different prognosis [11, 12].

Therefore, a decision tree model was constructed based on the clinical information of postoperative HCC patients from the Surveillance, Epidemiology, and End Results (SEER) database, and the survival benefit of chemotherapy was evaluated in high- and low-risk patients identified by this model. The present study may provide a new method and reference for the postoperative management of patients with HCC in the future.

## Material and methods

### Data acquisition and study design

**Fig. 1:**
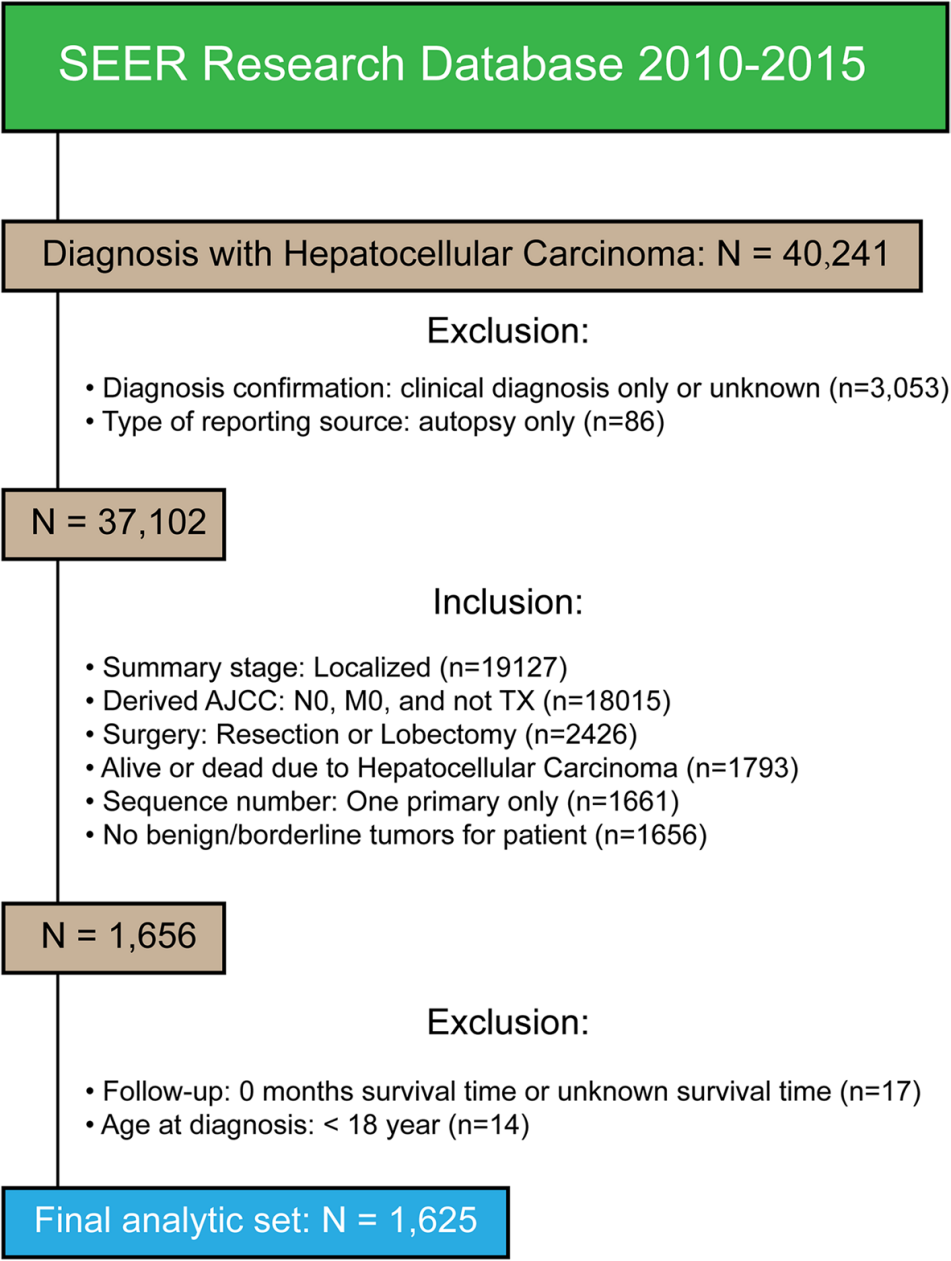
Data acquisition and the inclusion and exclusion criteria of patients

All patients diagnosed with HCC between January 2010 and December 2015 were downloaded from the SEER database (Fig. 1). We mainly wanted to study the prognosis of adult primary liver cancer with no lymph node involvement and no distant metastasis after hepatectomy. Inclusion criteria are as follows: patients who underwent resection or lobectomy; localized stage; AJCC staging N0, M0, and not TX; and alive or dead due to hepatocellular, there is only one primary tumor, and no benign or borderline tumors were present [13, 14]. Exclusion criteria are as follows: clinical diagnosis only or unknown, reporting source of autopsy only, survival time was 0 month or unknown survival time, and age at diagnosis < 18 [15–17]. Endpoint outcome of this study was 5-year cancer-specific death (CSD).

### Model construction and validation

One-thousand six-hundred twenty-five eligible patients with HCC were divided into training group and validation group with a 4:1 ratio using block randomization. Risk factors for 5-year CSD were determined by univariate and multivariate logistic analysis. Next, a risk prediction model for 5-year CSD of patients with HCC after surgery was established by using the classical decision tree method. The classical decision tree was based on binary output variables and predictor variables, and all variables input into analysis were optimized for binary classification. If it was a continuous variable, a cutoff value was selected for classification to maximize the purity of the two categories. The reliability of the model was evaluated by receiver operating characteristic curve (ROC). Optimal sensitivity and specificity were considered to determine the cutoff values to identify high- and low-risk patients. Validation group were used to verify the prediction performance of the model.

### Statistical analysis

The decision tree model was constructed by Orange3 software, and the rest results were analyzed by SPSS and R software [18]. Continuous variables were presented as mean ± SD and compared using *t*-test, and classified variables were compared using *χ*^2^ test. Logistic analysis was used for univariate and multivariate analyses. Decision tree method was used for model construction. Area under curve (AUC), F1 score, precision, and recall radio were used for model evaluation. missForest package was used for random interpolation after removing the variables with missing data > 30% [19]. The propensity score-matching (PSM) method was used to correct for significant differences in the sample sizes of the high- and low-risk groups. *P* < 0.05 was considered statistically significant.

## Results

### Subjects grouping and clinical characteristics

In accordance with the 4:1 rule, 1625 eligible patients were randomly divided into the training cohort (*n* = 1300) and the validation cohort (*n* = 325). There were differences in race and 5-year CSD between the two groups and no differences in age at diagnosis, gender, marital status, grade, AFP level, vascular invasion, tumor size, number of lesions, AJCC_T stage, and whether to receive chemotherapy (Table 1).

**Table 1:** Clinical characteristics of HCC patients (SEER 2010–2015)

Baseline variables	Training cohort (***N*** = 1300)	Internal testing cohort (***N*** = 325)	***P***
Vascular invasion			0.683a
No	1047	265	
Yes	253	60	
Tumor size (cm)			0.444a
< 2 cm	136	42	
2–5 cm	625	151	
> 5 cm	539	132	
AFP			0.96a
Negative	558	139	
Positive	742	186	
Marital status			0.76a
Married	796	196	
Others	504	129	
Lesion			0.528a
Single	1106	281	
Multiple	194	44	
Grade			0.884a
Well	307	73	
Moderately	679	168	
Poorly	283	77	
Undifferentiated	31	7	
Chemotherapy			0.613a
No	1153	285	
Yes	147	40	
Age at diagnosis			0.532a
18–45	85	16	
46–65	704	176	
> 65	511	133	
AJCC_T			0.64a
1	895	229	
2	310	77	
3	95	19	
Gender			0.079a
Women	360	106	
Man	940	219	
Race			**0.003b**
American Indian/Alaska Native	10	8	
Asian or Pacific Islander	514	123	
Black	222	41	
White	871	153	
**5-year CSD**			**0.006a**
**No**	**974**	**267**	
**Yes**	**326**	**58**	

*AFP*, α-fetoprotein

^a^Pearson’s *χ*^2^ test

^b^likelihood ratio

### Determination of independent risk factors

**Fig. 2:**
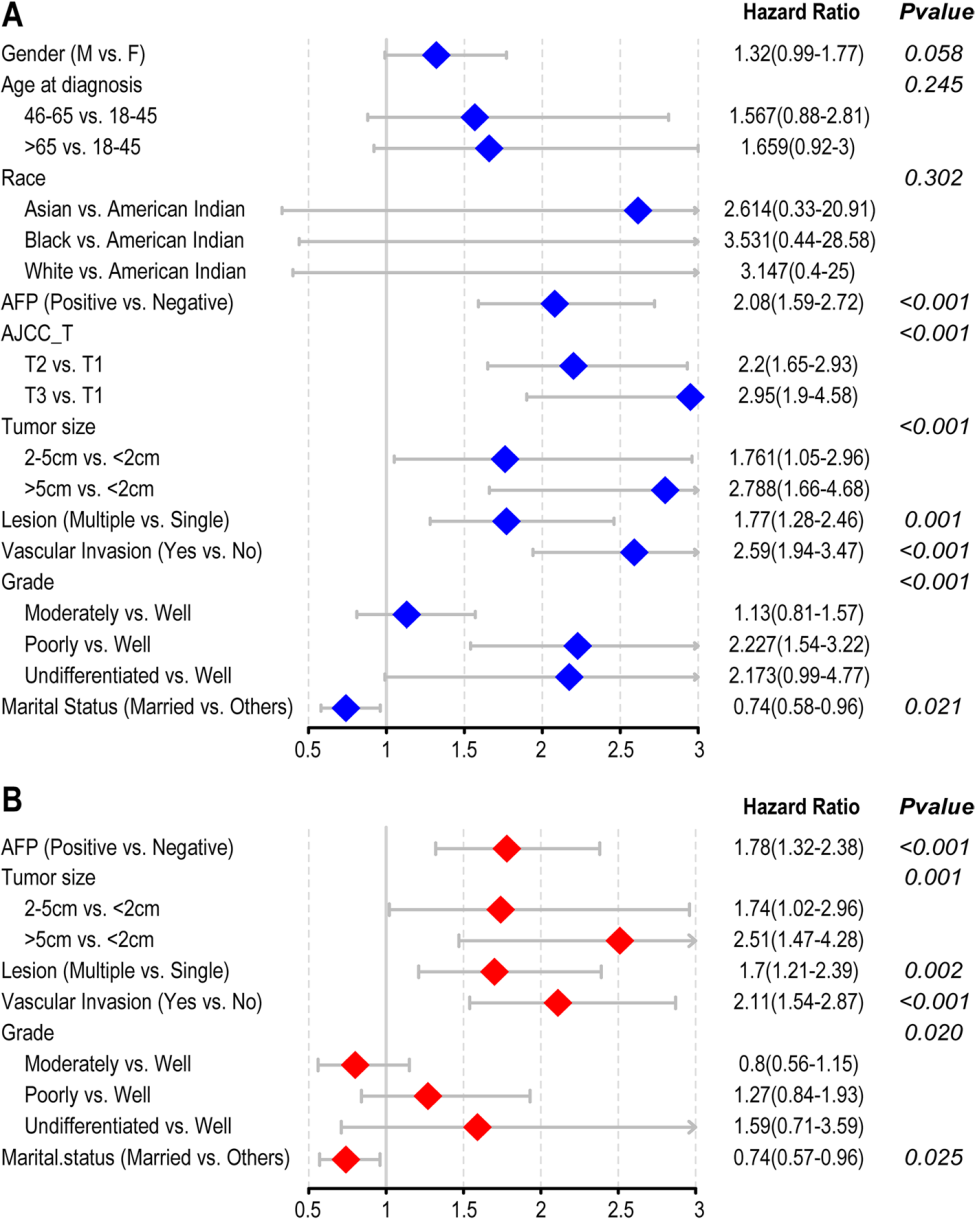
Univariate and multivariate analysis of variables with CSD. Univariate (**A**) and multivariate (**B**) logistic analysis for risk factor identification in the training group

Univariate (Fig. 2A) and multivariate (Fig. 2B) logistic analyses were conducted in the training group to obtain independent risk factors. Univariate analysis of the clinical parameters showed that marital status, grade, AFP level, vascular invasion, tumor size, number of lesions, and T stage were related to the 5-year CSD of patients. Multivariate analysis showed that marital status, grade, AFP, vascular invasion, tumor size, and number of lesions were independent risk factors for 5-year CSD of patients. We found that married was a good prognostic factor for HCC, and AFP-positive and vascular invasion suggested a poor prognosis. And the lower the degree of differentiation, the larger the tumor volume, and the more the number of tumors, the worse the prognosis.

### Construction and verification of decision tree model

**Fig. 3:**
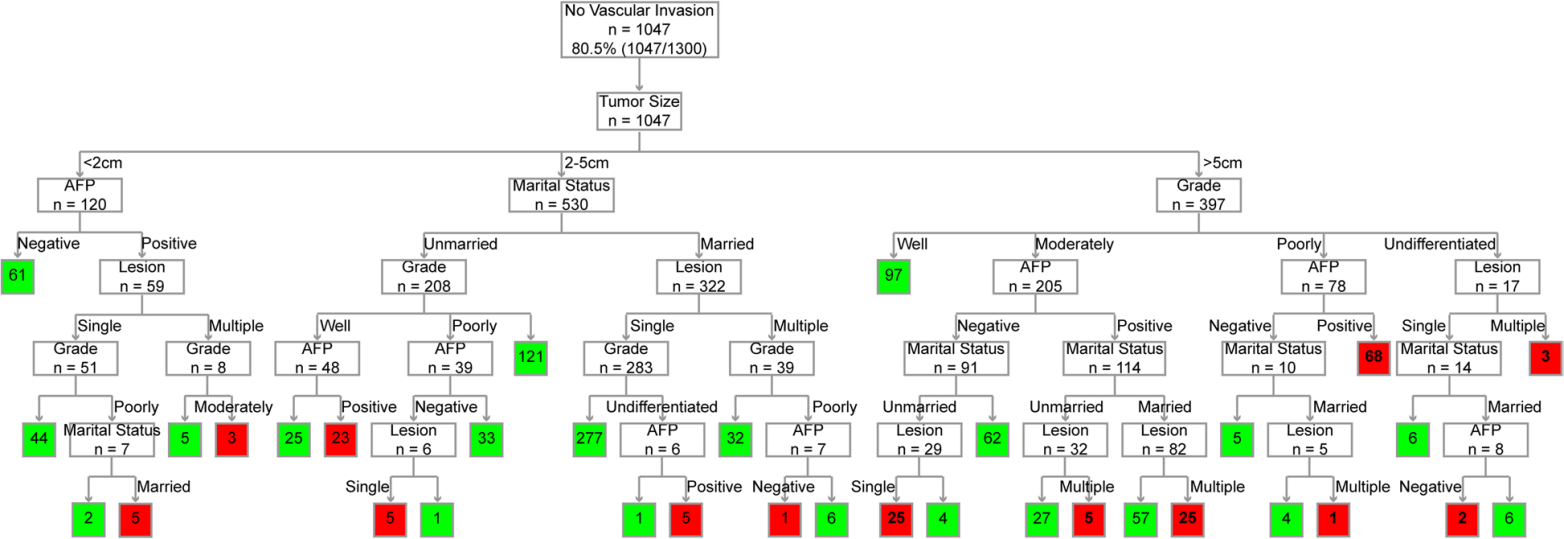
Decision tree model to predict 5-year CSD in HCC patients (without vascular invasion). Green represents low-risk; red represents high risk

**Fig. 4:**
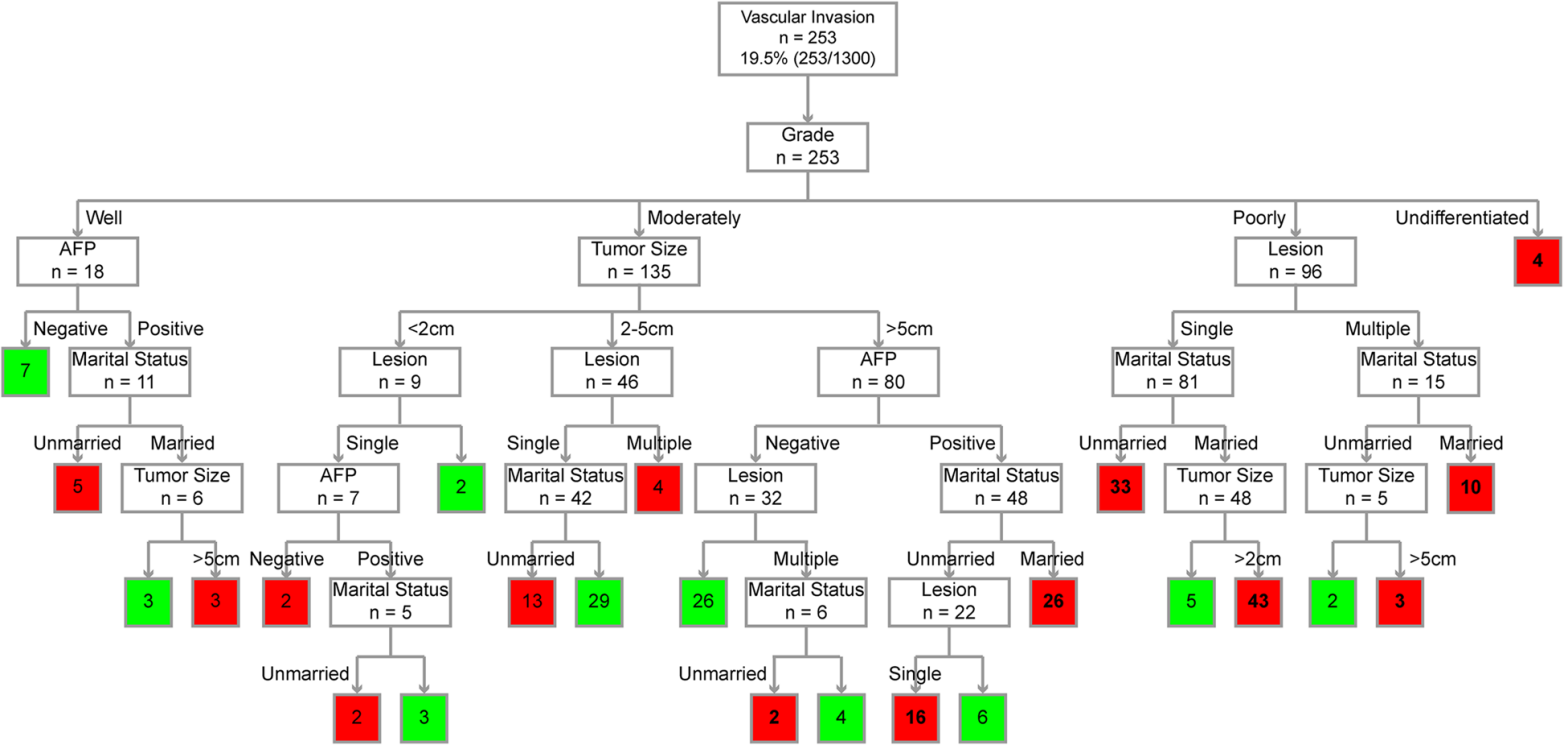
Decision tree model to predict 5-year CSD in HCC patients (with vascular invasion). Green represents low risk; red represents high risk

**Fig. 5:**
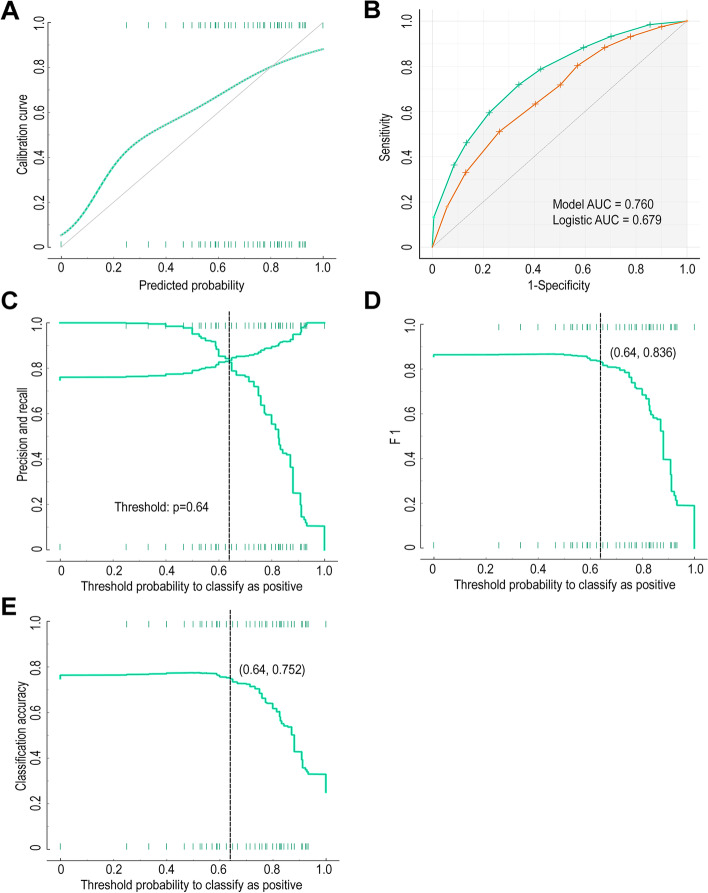
Evaluation results of the model in the training cohort. **A** The calibration curve of the model. **B** The ROC of the model and logistic regression. **C** The precision-recall curve determines that the threshold of the model is 0.64. **D** The F1 score of the model. **E** The classification accuracy of the model

The independent risk factors derived from multivariate logistic analysis of the training group were used to construct a risk prediction model for 5-year CSD using a decision tree algorithm. The model constructed is shown in Figs. 3 and 4. Figure 3 shows the results of classifying patients without vascular invasion using the decision tree model. One-hundred seventy-one (16.3%, 171/1047) patients without vascular invasion were at high risk of CSD for 5 years. It can be observed from the figure that tumor size > 5cm is a risk factor for 5-year CSD (32.5%, 129/397), and patients with poorly and undifferentiated stage are high-risk groups for 5-year CSD (77.9%, 74/95). Figure 4 shows the results of classifying patients with vascular invasion using the decision tree model. One-hundred sixty-six (65.6%, 166/253) patients with vascular invasion were at high risk of CSD for 5 years. Consistent with the above results, tumor size > 5cm (55%, 44/80) and poorly and undifferentiated stage (93%, 93/100) are the main risk factors for CSD 5 years after liver cancer surgery. Then, we calculated the calibration curve of the model and found that the model had good fitting ability (Fig. 5A). We compared the ROC (Fig. 5B) of decision tree and logistic regression and found that the decision tree model (*AUC* = 0.76) had stronger prediction ability than logistic regression (*AUC* = 0.679). Then, we determined the threshold (threshold = 0.64) of the model according to the precision and recall (Fig. 5C). Patients were classified as high (survival rate ≤ 0.64) and low risk (survival rate > 0.64) according to this threshold. We also calculated the F1 (*F1* = 0.836, Fig. 5D) and classification accuracy (classification accuracy = 0.752, Fig. 5E) of the model when the model threshold was 0.64. In the validation set, when the threshold was 0.64, AUC, classification accuracy, precision, recall, and F1 scores were 0.729, 0.757, 0.873, 0.824, and 0.848, respectively (Table 2). According to the model, all patients (*n* = 1625) with HCC undergoing surgery could be divided into two groups, of which 413 cases were high-risk group and 1212 cases were low-risk group (Additional file 1). These data suggested that the decision tree model had good prediction performance.

**Table 2:** Evaluation results of the model in the training and internal testing cohort

Cohort	AUC	Classification accuracy	F1	Precision	Recall
Training	0.76	0.752	0.836	0.83	0.842
Testing	0.729	0.757	0.848	0.873	0.824

### Effect of surgery combined with chemotherapy on high-risk and low-risk patients

To further explore the effect of surgery combined with chemotherapy on the prognosis of HCC patients, the high-risk group and low-risk group were further divided into two subgroups according to whether or not they had received chemotherapy. In the high-risk group, there was a significant difference in AFP between surgery alone and surgery combined with chemotherapy. In order to eliminate this confounding factor, we treated with PSM. After PSM correction, there was a significant difference in 5-year CSD between the two groups. The 5-year survival rate of patients treated with surgery alone was 15.5% (11/71), and that of patients treated with surgery combined with chemotherapy was 35.2% (25/71) (Table 3). In the low-risk group, there were significant differences in AFP, lesion, and grade between surgery alone and surgery combined with chemotherapy. We used PSM to eliminate these confounding factors. We found no difference in 5-year CSD between the two groups. These data suggested that surgery combined with chemotherapy can significantly improve the prognosis of HCC patients in the high-risk group, but it has no effect on the prognosis of HCC patients in the low-risk group (Table 4).

**Table 3:** Characteristics of high-risk HCC patients

Characteristics	Before PSM			After PSM		
	Without chemotherapy (***N*** = 342)	With chemotherapy (***N*** = 71)	***P***	Without chemotherapy (***N*** = 71)	With chemotherapy (***N*** = 71)	***P***
Vascular invasion			0.620a			0.867a
No	172	38		37	38	
Yes	170	33		34	33	
Tumor size (cm)			0.331a			0.174b
< 2 cm	12	4		1	4	
2–5 cm	93	14		21	14	
> 5 cm	237	53		49	53	
AFP			**0.011a**			1a
Negative	74	6		6	6	
Positive	268	65		65	65	
Marital status			0.071a			0.489a
Married	162	42		46	42	
Others	180	29		25	29	
Lesion			0.068a			0.462a
Single	266	48		52	48	
Multiple	76	23		19	23	
Grade			0.839b			0.722b
Well	32	6		6	6	
Moderately	126	25		19	25	
Poorly	168	38		43	38	
Undifferentiated	16	2		3	2	
5-year CSD			**0.001a**			**0.007a**
No	194	25		11	25	
Yes	148	46		60	46	

*PSM*, propensity score matching; *AFP*, α-fetoprotein

^a^Pearson’s *χ*^2^ test

^b^likelihood ratio

**Table 4:** Characteristics of low-risk HCC patients with AJCC 8th edition stages 1–3

Characteristics	Before PSM			After PSM		
	Without chemotherapy (***N*** = 1096)	With chemotherapy (***N*** = 116)	***P***	Without chemotherapy (***N*** = 112)	With chemotherapy (***n*** = 112)	***P***
Vascular invasion			0.873a			0.472a
No	997	105		104	101	
Yes	99	11		8	11	
Tumor size (cm)			0.388a			0.777a
< 2 cm	148	14		11	14	
2–5 cm	610	59		63	59	
> 5 cm	338	43		38	39	
AFP			**0.019a**			0.787a
Negative	570	47		49	47	
Positive	526	69		63	65	
Marital status			0.484a			0.265a
Married	716	72		76	68	
Others	380	44		36	44	
Lesion			**0.008a**			0.604a
Single	979	94		93	90	
Multiple	117	22		19	22	
Grade			**< 0.001b**			0.437b
Well	309	33		25	33	
Moderately	645	51		63	51	
Poorly	129	25		21	25	
Undifferentiated	13	7		3	3	
5-year CSD			0.306a			0.741a
No	928	94		88	90	
Yes	168	22		24	22	

*PSM*, propensity score matching; *AFP*, α-fetoprotein; *CSD*, cancer-specific death

^a^Pearson’s *χ*^2^ test

^b^likelihood ratio

## Discussion

The progress of surgical resection, ablation, and liver transplantation has improved the prognosis of HCC patients to some extent, but compared with other common human cancers, the long-term survival rate of HCC patients is still not ideal due to the high recurrence rate and lack of effective adjuvant therapy [20, 21]. Therefore, we must carry out hierarchical management and targeted treatment for postoperative patients with different risk levels in order to improve the long-term survival rate of patients with liver cancer. In this study, we found that tumor size, vascular invasion, AFP level, and number of lesions were independent risk factors for 5-year CSD through univariate and multivariate logistic regression analysis. Married was a good prognostic factor for HCC, and AFP-positive and vascular invasion suggested a poor prognosis. And the lower the degree of differentiation, the larger the tumor volume, and the more the number of tumors, the worse the prognosis. Previous studies have shown that tumor size, vascular invasion, AFP level, and number of lesions may affect the prognosis of patients with HCC, which is consistent with the results of this study [22–24]. Interestingly, in this study, it was found that marital status was also an independent risk factor for 5-year CSD. This is in keeping with previous reports that married patients had better 5-year HCC cause-specific survival than did unmarried patients (46.7% vs 37.8%) [25]. Marital status is an important prognostic factor for survival in patients with HCC treated with surgical resection.

There have also been previous reports on the postoperative prognosis model of HCC. Shim et al. established the survival nomogram of postoperative HCC patients (*AUC* = 0.66) [26]. This study also constructs a logistic regression model (*AUC* = 0.679). In contrast, the decision tree model (*AUC* = 0.760) in this study has better prediction performance. It seems to have greater clinical application potential. In the present study, vascular invasion, tumor size, and poor differentiation were the main risk factors for 5-year CSD in HCC patients after surgery, which is in keeping with previous studies [27, 28]. The prognosis of patients with vascular invasion, tumor size > 5cm, or poorly stage is poor. The decision tree prediction model in this study can accurately predict the high-risk group of patients with 5-year CSD after HCC surgery, help to realize patient-specific early diagnosis and treatment, and further improve the prognosis of HCC patients.

In recent years, some studies have found that surgical resection of HCC combined with chemotherapy can improve the postoperative survival rate [29–31]. However, there are no clinical guidelines recommending the routine use of surgery combined with chemotherapy for HCC patients because the beneficiaries are still uncertain. In this study, for the high-risk and low-risk patients divided based on the decision tree model, in the high-risk patients, the prognosis was significantly improved after surgery combined with chemotherapy, while in the low-risk patients, there was no significant change in CSD 5 years after surgery combined with chemotherapy. This means that the prognostic model established in this study can provide a reference for guiding the management of postoperative adjuvant chemotherapy.

The data source of this study is SEER database, which is an important resource for practical research in oncology. One-thousand six-hundred twenty-five HCC patients with complete clinical data were included. The characteristic distribution of the data is normal, and the model has good prediction performance in both training set and verification set, which provides a sufficient and reliable basis for further clinical application. However, this study also has some limitations. Because this study is based on a public database, the collection of clinical data is limited by the items provided in the data set, and it is impossible to explore more possible prognostic factors. In addition, the prognostic risk prediction model constructed in this study still needs external validation to further confirm its effectiveness.

## Conclusions

The 5-year CSD prediction model based on decision tree algorithm provides accurate prediction information. The high-risk patients determined by the prediction model may benefit from the 5-year survival after surgery combined with chemotherapy. The prediction model is expected to provide reference for postoperative management of patients with HCC in the future.

## Supplementary Information

**Additional file 1.** Clinical Characteristics of 1625 eligible patients included in this study

## Data Availability

Publicly available datasets were analyzed in this study. This data can be found here: Surveillance, Epidemiology, and End Results (SEER) database (https://seer.cancer.gov/).
